# Right-sided diaphragmatic eventration: A rare entity

**DOI:** 10.4103/0970-2113.48898

**Published:** 2009

**Authors:** A. P. Kansal, Vishal Chopra, A. S. Chahal, Charanpreet S. Grover, Harpreet Singh, Saurabh Kansal

**Affiliations:** *Department of Chest and TB, Govt. Medical College, Patiala, India*

**Keywords:** Diaphragm, eventration, sniff test, fluoroscopy

## Abstract

Eventration of the diaphragm is an abnormal elevation of the dome of diaphragm. It is a condition in which all or part of the diaphragm is largely composed of fibrous tissue with only a few or no interspersed muscle fibers. It can be complete or partial. Complete eventration of the right diaphragm, as seen in this adult patient, is relatively rare.

## INTRODUCTION

Eventration of diaphragm is a congenital anomaly consisting of failure of muscular development of part or all of one or both hemidiaphragms.^1^

Clinically, eventration of diaphragm refers to an abnormal elevation of one leaf of an intact diaphragm as a result of paralysis, aplasia, or atrophy of varying degrees of muscle fibers.^2^ In some cases, it may be difficult or impossible to distinguish from diaphragmatic paralysis.^1^ Complete eventration almost invariably occurs on the left side^3^ and is rare on the right.

We report a case of 67-year-old female with right-sided eventration.

## CASE HISTORY

A 67-year-old female presented to us with complaints of cough with expectoration and on and off fever since one year.

General physical examination was normal. The patient was afebrile during her stay in the hospital. Chest examination revealed decreased movements on right side in inframammary, infra-axillary, and infrascapular areas. Tactile vocal fremitus was decreased and note was impaired on the right side. Breath sounds were decreased in the right inframammary, infra-axillary, and infrascapular areas.

**Figure 1:**
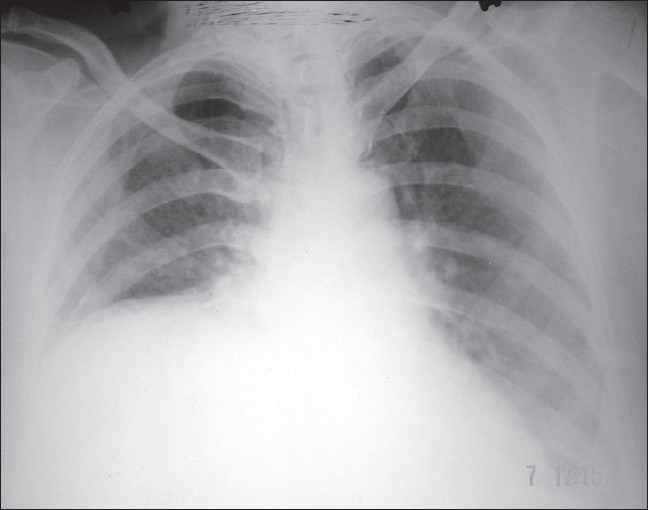
Chest X-ray posteroanterior view showing eventration of right dome of diaphragm

**Figure 2:**
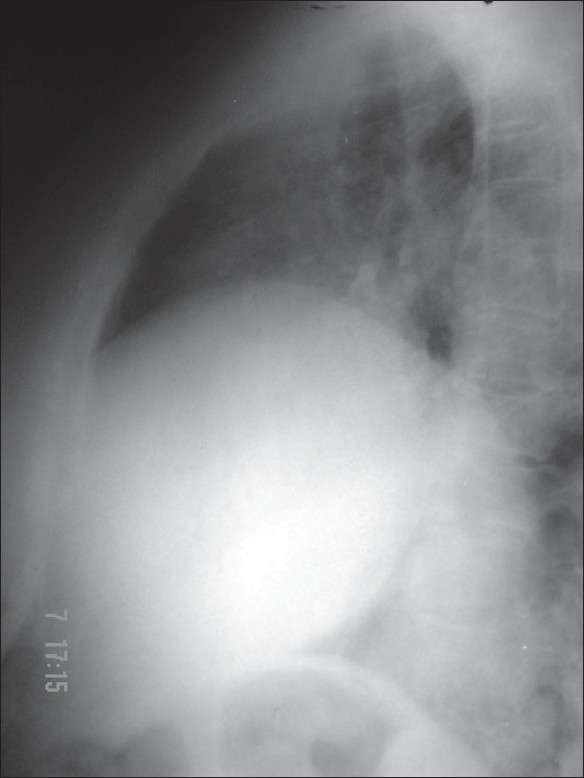
Chest X-ray right lateral view showing eventration of right dome of diaphragm

**Figure 3:**
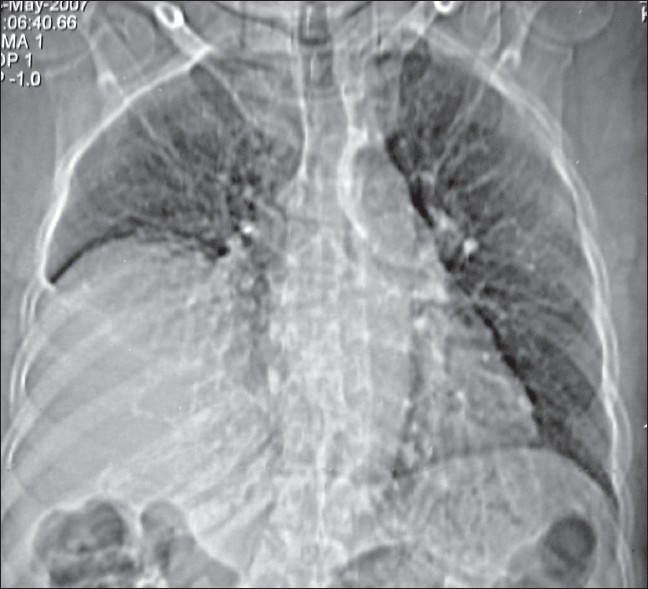
CT scan of the chest showing complete eventration of right dome of diaphragm

Laboratory investigations were within normal limits. Chest X-ray showed a homogenous opacity in the right lower zone. The upper margin of the opacity was sharp and had a contour of a diaphragm on posterioanterior (PA) view which was further confirmed with a right lateral view [Figure 1, Figure 2]. This increased our suspicion of raised dome of diaphragm. Fluoroscopy was performed with the patient in erect and supine position. There was absence of paradoxical movements of the right diaphragm which was confirmed on sniff test. Ultrasonography did not show any abnormality except markedly restricted movements of the right diaphragm and lagging during respiration. CT scan of the chest revealed that right hemidiaphragm was raised as compared to the left side with a smooth contour [Figure 3]. There was no herniation of abdominal contents into the thoracic cavity. The mediastinal structures were well opacified with i.v. contrast and appeared normal in outline. There was no evidence of any mediastinal lymph node enlargement. With these radiological findings, a diagnosis of eventration of right diaphragm was made.

## DISCUSSION

Eventration of the diaphragm is a condition in which all or part of the diaphragm is largely composed of fibrous tissue with only a few or no interspersed muscle fibers. It is usually congenital but may be acquired.^4^

Complete eventration of diaphragm invariably occurs on the left side but partial eventration of the diaphragm occurs virtually on the right side.^3^ In this case, the complete eventration of diaphragm was seen on the right side which is a rarity. Eventration of diaphragm is generally asymptomatic in adults and is discovered incidentally on normal screening of chest X-ray as was in the present case. Symptoms may be present in obese patients as a result of raised intra-abdominal pressure. These symptoms, related to gastrointestinal tract, respiratory embarrassment, and rarely cardiac dysfunction, have been attributed to the anomaly.^5^

Elevation of diaphragm can also be attributed to interruption of phrenic nerve by neoplasm or surgical resection. In adults it is very difficult or impossible to distinguish it from diaphragmatic paralysis. These entities can be distinguished radiologically. In adults the diagnosis of diaphragmatic eventration can usually be made on standard PA and lateral chest films.^6^ In the PA projection, the elevated diaphragm forms a round unbroken line arching from the mediastinum to the costal arch. Conventional chest radiography has been found to be a useful modality for assessment of the functional status of an elevated diaphragm as the evaluation of the shape of an elevated diaphragm may preclude the need for fluoroscopic sniff test to determine diaphragmatic paralysis.^7^

Flouroscopy is considered the most reliable way to document diaphragmatic paralysis and the sniff test is necessary to confirm that abnormal hemidiaphragm excursion is due to paralysis rather than unilateral weakness.^8^ Ultrasonography can help in establishing the diagnosis of partial eventration and in distinguishing it from diaphragmatic nerve interruption. The diaphragm can be seen as a continuous thin layer above the elevated abdominal viscera and on real-time ultrasound the abnormal region can be seen to move downward with the normal portion although it may show a slight lag in its inspiratory excursion.^9^

The radiological sight of complete eventration is identical to that diaphragmatic paralysis. In some cases, however, there is no way of knowing whether elevation is caused by congenital absence of muscle or by phrenic paralysis.^10^

Asymptomatic patients are managed conservatively but patients with symptoms require surgery. Paradoxical movements suggest complete paralysis and if symptomatic, is a strong indication of surgery.

In the present case, a diagnosis of eventration of diaphragm was made based on radiological findings.
